# Supplementary material for: Combination Treatment of Locoregionally Aggressive Granulomatosis with Polyangiitis and Cranial Base Infiltration
Source: Brain Sci. 2023 Jul 29;13(8):1140. doi: 10.3390/brainsci13081140 (PMC10452321; doi:10.3390/brainsci13081140)
Supplement: Supplementary file 1 [file brainsci-13-01140-s001.zip › brainsci-2483106-supplementary.pdf]

Author	Other clinical manifestations	Surgical/local intervention	cANCA	The time between diagnosis of GPA and skull-base infiltration	Initial treatment	Treatment of inflammatory pseudotumor in the base of the skull	Outcome	Localization
Carpentier et al. [42]	Headache, pulmonary mass	No data	Positive	At the time of diagnosis	No data	No data	No data	Temporal bone pseudotumor
Sharma et al. [43]	Facial palsy, deafness, lung infiltrations	No	Positive	A the time of diagnosis	MP 60mg/d	No additional treatment	Resolution of inflammatory mass	Patchy, predominantly hypointense lesions in the central and posterior skull base adjoining the clivus and jugular foramen bilaterally
Bernat et al. [44]	Insomnia, headache, arthritis<br>excavated lesion of the right lung	Resection of the frontal lesion	Positive	A the time of diagnosis	MP 40mg/d<br>AZA 150mg/d	No additional treatment	Resolution of inflammatory mass	Olfactory groove extending to the right cribriform plate and a bilateral enhancement of the dura at the front-olfactory zone
Andrews et al. [45]	Left hemifacial pain and paresthesia, dysphagia, hoarseneshemoptysis, proteinuria with hematuria	No	Positive	A the time of diagnosis	CYC and GCs (no additional data)	No additional treatment	Chronic paresis of left cranial nerves IX, X, XL, and XII	Nasopharyngeal mass that extended through the posterior and lateral nasopharyngeal walls following the course of the eustachian tube
Qureshi et al. [46]	Severe bitemporal pain, cranial nerves IV, VI, and X palsies	Sinus surgery	Positive	A the time of diagnosis	MP pulses (no additional data) and RTX 4x375mg/m2	No additional treatment	Chronic paresis of cranial nerves VI and X	No data
Kim et al. [47]	Pain and facial swelling in the right cheek, cranial nerve V palsy	No	Negative	A the time of diagnosis	MMF and GCs (no additional data)	No additional treatment	Partial resolution of symptoms	Infratemporal mass, pterygopalatine fossae extending into the inferior right orbital fissure along the maxillary division of the trigeminal nerve
Case 1	Myalgia, nasal crusting, and pain with hearing loss in the left ear. Cranial nerves VI, VII, VIII, X, and XI palsies with cerebellar symptoms and a severe headache.	Intratympanic 4mg dexamethasone	Positive	After 3 months	CYC (3000mg)<br>MP 40mg/d	RTX 4x375mg/m2 and CYC (1700mg) with pulses of MP (4x1g/d)	Resolution of symptoms, partial resolution of inflammatory mass in MR and CT	In text and figures
Case 2	Pauci-immune glomerulonephritis with kidney failure (involving 80% of glomeruli in renal biopsy), involvement of the upper respiratory tract with the destruction of paranasal sinuses, and cranial with nerve palsies	Intratympanic 4mg dexamethasone	Positive	After 3 years	Induction therapy: MP pulses 5000mg/d,<br>CYC 1100mg,<br>RTX 4x375mg/m2<br><br>Maintenance therapy: GCs (prednisone, 5-30mg/d) with MTX 25mg/week, later MMF 2g/d	CYC (6000mg, lifetime dose 17000mg) and RTX 4x375mg/m2	Improvement of symptoms, stabilization of inflammatory mass in MR and CT	In text and figures
Case 3	Loss of hearing, severe otalgia, nasal crusting, and mild cerebellar symptoms.	Intratympanic 4mg dexamethasone	Positive	After 1 year	Induction therapy: CYC (5600mg)<br>MP (3x500mg)<br><br>Maintenance therapy: prednisone, 30-60 mg/d<br>MTX 25mg/week, later AZA 150mg/d	MP pulses (3x500mg/d) followed by GCs (prednisone 1mg/kg/d)<br>MMF (2g/day) and RTX 4x375mg/m2	Improved hearing and withdrawal of the temporal bone changes in CT	In text and figures

Supplementary Table S1. Granulomatosis with polyangiitis with cranial base infiltration cases. AZA – azathioprine, CYC – cyclophosphamide, GCs – glucocorticoids, MMF – mycophenolate, MP – methylprednisolone, MTX – methotrexate, RTX – rituximab,
